# A Rapidly Tunable Laser System for Measurements of NH_2_ at 597 nm Behind Reflected Shock Waves

**DOI:** 10.3390/s24247920

**Published:** 2024-12-11

**Authors:** Sean Clees, Spencer C. Barnes, Taylor M. Rault, Christopher L. Strand, Ronald K. Hanson

**Affiliations:** Department of Mechanical Engineering, Stanford University, Stanford, CA 93405, USA; spencer.c.barnes@stanford.edu (S.C.B.); trault@stanford.edu (T.M.R.); cstrand@stanford.edu (C.L.S.); rkhanson@stanford.edu (R.K.H.)

**Keywords:** laser absorption spectroscopy, visible lasers, second-harmonic generation, ammonia, shock tubes

## Abstract

Distributed feedback lasers, which feature rapid wavelength tunability, are not presently available in the yellow and orange spectral regions, impeding spectroscopic studies of short-lived species that absorb light in this range. To meet this need, a rapidly tunable laser system was constructed, characterized, and demonstrated for measurements of the NH_2_ radical at 597.4 nm. The system consisted of three main parts: (1) a distributed feedback diode laser operating at 1194.8 nm, (2) a fiber-coupled optical amplifier, and (3) a periodically poled lithium niobate (PPLN) waveguide for second-harmonic generation. A phase-matching optical frequency bandwidth of 118 GHz and a second-harmonic generation efficiency of 109%/W were determined for the PPLN waveguide, and the intensity and wavelength stability of the system were measured. The rapid-tuning capabilities of the laser system were characterized to explore its potential for use in scanned-direct absorption and wavelength modulation spectroscopy experiments. The feasibility of scanned-direct absorption up to a scan rate of 900 kHz and wavelength modulation spectroscopy at modulation frequencies up to 800 kHz were demonstrated. Finally, the system was deployed in a series of shock tube experiments in which the concentration of NH_2_ radicals was measured during the decomposition of NH_3_ behind reflected shock waves.

## 1. Introduction

Rapidly tunable, continuous-wave (CW) laser sources for the generation of narrow-linewidth light are desirable for applications in high-resolution laser absorption spectroscopy (LAS) in transient environments [1]. Distributed feedback (DFB) lasers are often used for such applications, as they feature narrow linewidths and can be rapidly tuned in wavelength by modulating the laser injection current [2,3,4]. In the near- and mid-infrared spectral regions, DFB diodes, interband cascades, and quantum-cascade lasers are commercially available [1]. In the blue spectral range, DFB lasers based on gallium nitride gain media are becoming available [5]. However, DFB sources for the generation of yellow and orange light are not yet commercially available. This has frustrated efforts to conduct high-temperature spectroscopic studies of short-lived species that absorb in this range, including NH_2_, a radical species key to the pyrolysis and oxidation of NH_3_ that absorbs in the vicinity of 597.4 nm [6].

Historically, dye and solid-state lasers have been used to generate narrow-linewidth, CW visible light [7]. However, the reliance of such systems on optical cavities typically precludes the rapid and broad mode-hop-free tuning of the output wavelength [8]. To rectify this, some studies have employed resonant galvanometer-based modulation of a dye laser cavity, which is limited in tuning range and rate by the physical motion of the length-tuning element [8]. Following these early studies, the development of quasi-phase-matched (QPM) crystals with large $\chi^{\left(2\right)}$ nonlinearity enabled the generation of milliwatt levels of visible light via second-harmonic generation (SHG) in a single-pass configuration [9,10,11]. Lasers employing QPM crystals and waveguides have been demonstrated for fixed-wavelength operation at 488 nm [12], 578 nm [13], 495 nm [14], and other wavelengths [15,16,17,18,19], but the rapid wavelength-tuning capabilities of such systems and their application to LAS studies have yet to be explored.

To this end, the present work describes the design, characterization, and demonstration of a master oscillator power amplifier (MOPA) laser system for the generation of rapidly tunable light in the yellow-orange spectral region at 597.4 nm. The system is based on a DFB diode laser (DFB-DL) operating at 1194.8 nm. The DFB-DL is amplified by a fiber-coupled booster optical amplifier (BOA) for efficient SHG in a single pass through a periodically poled lithium niobate waveguide (PPLN-WG). The single-pass configuration preserves the desirable mode-hop-free rapid-tuning capabilities of the pump DFB-DL in the visible output. The long-term stability of the system was characterized for fixed-wavelength operation, and the tuning characteristics were determined for both scanned-direct absorption and wavelength modulation spectroscopy applications. Finally, the system was demonstrated for measurements of NH_2_ during NH_3_ pyrolysis experiments in a shock tube using a variety of LAS techniques. This multifaceted demonstration serves to illustrate the versatility of frequency-doubled DFB-DL systems for high-resolution spectroscopic studies of short-lived species and paves the way for the development of future systems at shorter wavelengths, potentially into the ultraviolet range.

## 2. Background

### 2.1. Quasi-Phase-Matched Second-Harmonic Generation

The generation of visible light in the present work relies on QPM SHG in a PPLN-WG. For efficient SHG to occur in a medium with appreciable $\chi^{\left(2\right)}$ nonlinearity, two criteria must be satisfied. First, energy must be conserved such that $\nu_{2}=2\nu_{1}$, where $\nu_{2}$ [cm^−1^] is the optical frequency of the SH light and $\nu_{1}$ [cm^−1^] is the optical frequency of the pump light [20]. Second, momentum must be conserved, leading to the first-order phase-matching condition given in Equation (1) derived from the wave vectors of the involved photons [21,22]:

(1) $$\Delta k=2\pi \left(2n_{1}\left(T\right)\nu_{1}-n_{2}\left(T\right)\nu_{2}-\frac{1}{\Lambda (T)}\right),$$

where Δk is the wave vector mismatch; $n_{1}$ and $n_{2}$ are the indexes of refraction at optical frequencies 1 and 2, respectively; *T* is the crystal temperature; and Λ [cm] is the poling period of the QPM crystal. Maximum SHG efficiency occurs when Δk=0, which can be achieved by adjusting the temperature of the QPM medium. In general, under minimal depletion of the pump laser, the efficiency of the SHG process varies as sinc^2^(Δk) [20], giving rise to a QPM bandwidth in temperature space (at a fixed optical frequency) and optical frequency space (at a fixed temperature). For Δk=0, the output SHG power from a QPM medium, $P_{2}$ [mW], can be expressed as per Equation (2) [23]:

(2) $$P_{2}=P_{1}\tanh^{2}\left(\sqrt{\eta P_{1}}\right),$$

where $P_{1}$ [mW] is the input pump power and η is the conversion efficiency.

### 2.2. Laser Absorption Spectroscopy

Laser absorption spectroscopy is a proven technique for quantitative, non-intrusive, and time-resolved measurements of chemical species in gas phase [1]. At its core, LAS relies on the Beer–Lambert relation, which relates the transmitted ($I_{t}$) and incident ($I_{0}$) intensity of light at optical frequency ν [cm^−1^], as given in Equation (3) [24]:

(3) $$-\ln{\left(\frac{I_{t}}{I_{0}}\right)}_{\nu }=\alpha_{\nu }=P\chi L\sum_{i}S_{i}\left(T\right)\phi_{\nu ,i}=k_{\nu }P\chi L,$$

where $\alpha_{\nu }$ is the absorbance at frequency ν, *P* [atm] is the pressure, χ is the absorbing species mole fraction, *L* [cm] is the path length, $S_{i}$ [cm^−2^·atm^−1^] is the linestrength of transition *i*, *T* [K] is the gas temperature, $\phi_{\nu ,i}$ [cm] is the normalized absorption lineshape of transition *i* at optical frequency ν, and $k_{\nu }$ [cm^−1^·atm^−1^] is the absorption coefficient. A variety of LAS techniques have been developed for various applications. In fixed-direct absorption (FDA), the simplest implementation of LAS, the laser wavelength is fixed near the peak of a spectroscopic transition or group of transitions. The species mole fraction is thus calculated directly from the measured absorbance based on knowledge of the linestrength and lineshape or absorption coefficient of the target feature [24]. In scanned-direct absorption (SDA), the laser wavelength is periodically scanned over the entirety of an absorption feature, and the measured absorbance can be integrated to remove dependence on the lineshape, as expressed in Equation (4) [24]:

(4) $$A_{i}=P\chi LS_{i}\left(T\right),$$

where $A_{i}$ [cm^−1^] is the integrated area of transition *i*. Additionally, a lineshape profile can be fit to the measured absorbance spectrum to extract quantities of interest such as pressure [24]. Lineshapes are commonly modeled as Voigt profiles, which are described by Doppler width, $\Delta \nu_{D}$ [cm^−1^], and collisional width, $\Delta \nu_{C}$ [cm^−1^], as given in Equations (5) and (6) [24]:

(5) $$\Delta \nu_{D}=7.1623\cdot 10^{-7}\nu_{0}{\left(\frac{T}{M}\right)}^{1/2},$$

(6) $$\Delta \nu_{C}=P\cdot \sum_{j}\chi_{j}2\gamma_{j}\left(T\right),$$

where $\nu_{0}$ [cm^−1^] is the transition linecenter, M [g·mol^−1^] is the molar mass of the absorbing molecule, and $2\gamma_{j}\left(T\right)$ [cm^−1^·atm^−1^] is the collisional broadening coefficient of the target transition for a particular collision partner, *j*. Values for $2\gamma_{j}\left(T\right)$ are often described by using a power law, in which they are characterized by a value at a reference temperature, $2\gamma_{j}\left(T_{0}\right)$, and a temperature-dependence exponent, $n_{j}$, as per Equation (7) [24]:

(7) $$2\gamma_{j}\left(T\right)=2\gamma_{j}\left(T_{0}\right){\left(\frac{T_{0}}{T}\right)}^{n_{j}}$$

Many LAS-based laser sensors have also been developed for use in practical systems outside the laboratory, where issues such as thermal emission, particle scattering, and window fouling can corrupt measurements [1]. Several advanced LAS techniques have been developed to combat such noise sources. These techniques typically involve the rapid modulation of laser optical frequency and/or intensity and the subsequent lock-in amplification of resulting high-frequency signals. In intensity modulation spectroscopy (IMS), only the intensity of the laser is appreciably modulated at a frequency $f_{m}$ [Hz], and lock-in amplification at that frequency is used to extract absorption information [25,26]. In wavelength modulation spectroscopy (WMS), both intensity and optical frequency are simultaneously modulated, generating harmonics at $2f_{m}$, $3f_{m}$, etc., which are sensitive to absorption [27]. Often, WMS measurements are conducted by modeling the laser optical frequency and intensity as periodic functions of time, *t*, as per Equations (8) and (9), respectively, and using these models to simulate the expected WMS signals to which measured signals can be compared [27].

(8) $$\nu \left(t\right)=\bar{\nu }+a_{1}\cos\left(2\pi f_{m}t\right)$$

(9) $$I_{0}\left(t\right)=\bar{I_{0}}\left[1+i_{1}\cos\left(2\pi f_{m}t+\phi_{1}\right)+i_{2}\cos\left(4\pi f_{m}t+\phi_{2}\right)\right]$$

In Equations (8) and (9), $\bar{\nu }$ [cm^−1^] is the average laser optical frequency; $a_{1}$ [cm^−1^] is the linear frequency amplitude; $\bar{I_{0}}$ is the average laser intensity; $i_{1}$ and $\phi_{1}$ are the linear intensity amplitude and phase shift, respectively; and $i_{2}$ and $\phi_{2}$ are the nonlinear amplitude and phase shift, respectively. Further details on FDA, SDA, IMS, and WMS are widely available in the literature [1,24,28,29].

### 2.3. NH_2_ Spectroscopy

**Figure 1:**
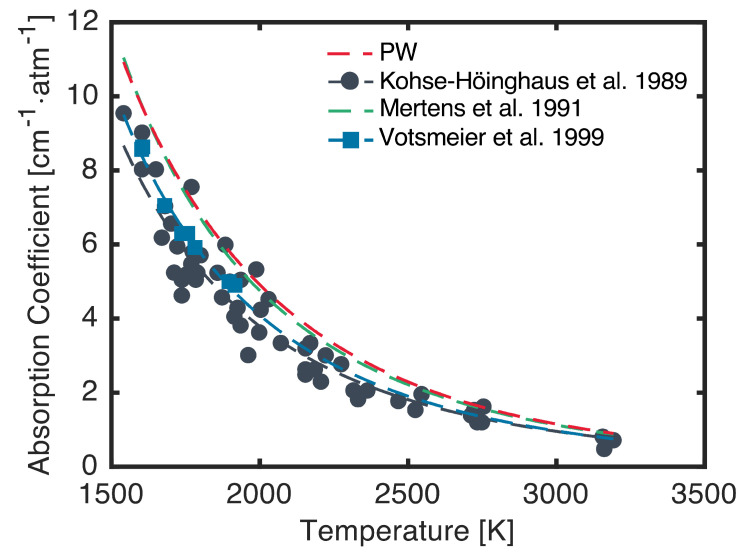
Absorption coefficient measurements (points) and correlations/calculations (lines) for the target NH_2_ feature at 16,739.90 cm^−1^ diluted in argon. Calculation from the present work (PW; red) was performed at a pressure of 1 atm. Measurements from Kohse-Höinghaus et al. [6] (black) were collected at various pressures between 0.5 atm and 1.2 atm, and the respective correlation is valid near 1 atm. Measurements and correlation from Votsmeier et al. [35] (blue) were scaled to a pressure of 1 atm by using the pressure scaling reported therein. Correlation from Mertens et al. [34] (green) is valid near 1 atm.

Though the architecture used in the present work is broadly applicable, the present system was designed to target an absorption feature of NH_2_ near 597.4 nm. The NH_2_ radical is a key intermediate species in the pyrolysis and oxidation of NH_3_, which is a potential zero-carbon fuel and hydrogen carrier [30,31]. The target absorption feature is the $\Sigma^{P}Q_{1,N}$(7) doublet in the ${\tilde{A}}^{2}A_{1}\left(090\right)\leftarrow {\tilde{X}}^{2}B_{1}\left(000\right)$ system and is located at 16,739.90 cm^−1^ (597.375 nm). This feature has been targeted previously for quantitative measurements of NH_2_, and several studies have reported absorption coefficient, oscillator strength, and broadening measurements for the doublet [6,32,33,34,35]. Notably, these studies predominantly used dye lasers to target this transition, and only one study performed rapid-tuning experiments by using a galvanometer-tuned dye laser [6]. Absorption coefficient measurements and correlations available in the literature are shown in Figure 1.

The most extensive measurements were performed by Kohse-Höinghaus et al. [6], who generated NH_2_ behind reflected shock waves via pyrolysis and laser photolysis of NH_3_ and measured the linecenter absorption coefficient, average oscillator strength, doublet splitting, doublet transition ratio, and argon broadening coefficient for the feature. These absorption coefficient measurements and the resulting correlation are shown in black in Figure 1. Davidson et al. [33] later targeted the same NH_2_ absorption feature in a series of NH_3_ pyrolysis measurements but found that an oscillator strength approximately 30% higher than that measured by Kohse-Höinghaus et al. [6] more accurately reproduced their data. Mertens et al. [34] then published a correlation based on this observation, which is shown in green in Figure 1. Subsequently, Votsmeier et al. [35] reported additional absorption coefficient and oscillator strength measurements by using methylamine pyrolysis as a precursor for NH_2_ radicals. The oscillator strength measurements therein corroborated the findings of Davidson et al. [33]. However, the absorption coefficient measurements and correlation reported by Votsmeier et al. [35], shown in blue in Figure 1, are more closely in line with the measurements reported by Kohse-Höinghaus et al. [6]. The source of this discrepancy between Votsmeier et al.’s [35] absorption coefficient and oscillator strength measurements is unclear. However, due to the agreement between Davidson et al. [33] and Votsmeier et al. [35] on the value of the oscillator strength, Votsmeier et al.’s [35] value was used in this work. Further validation of the oscillator strength of the target doublet will be the subject of future experiments.

For the absorption coefficient and linestrength values necessary for the demonstration of the present laser system, calculations were performed as described by Kohse-Höinghaus et al. [6]. Molecular constants, vibrational frequencies, and term energies for the computation of the Boltzmann fractions of the absorbing states were taken from Kohse-Höinghaus et al. [6], Green and Miller [32], and Dressler and Ramsay [36]. The doublet splitting, doublet transition ratio, and argon broadening coefficient were taken from Kohse-Höinghaus et al. [6]. As mentioned, the average oscillator strength of the doublet was taken from Votsmeier et al. [35]. The resulting peak absorption coefficient calculated over a range of temperature is shown in red in Figure 1 at a pressure of 1 atm, assuming the feature is entirely broadened by argon. These calculations closely match the correlation used by Mertens et al. [34].

## 3. System Architecture

**Figure 2:**
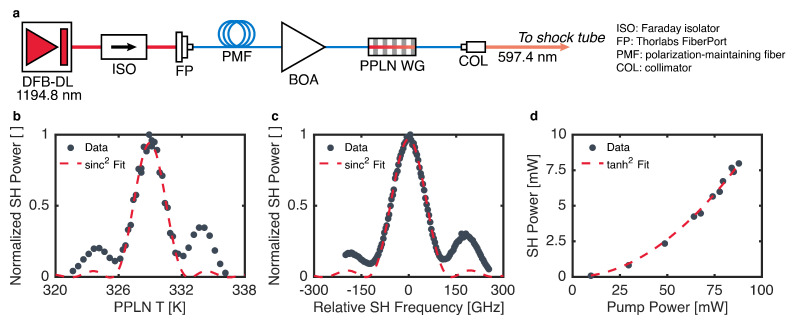
(**a**) Schematic of the laser system developed in this work. Measured power (points) and theoretical best-fit curves (lines) for determination of QPM temperature bandwidth (**b**), QPM frequency bandwidth (**c**), and SHG conversion efficiency (**d**).

A schematic of the laser system developed in this work is shown in Figure 2a. The system employed a DFB-DL as the pump laser operating near 1194.8 nm (Nanoplus, Meiningen, Germany), driven by a combination current/temperature controller (ILX Lightwave, Bozeman, MT, USA, LDC-3916372). For rapid-tuning operation, the injection current of the DFB-DL was modulated by using a digital function generator with a bandwidth of 15 MHz (SRS, Sunnyvale, CA, USA, DS340). The DFB-DL was fiber-coupled into a polarization-maintaining (PM) fiber patch cable (Thorlabs, Newton, NJ, USA, P3-980PM-FC-1) by using a five-axis FiberPort (Thorlabs PAF2-7C). A free-space Faraday isolator (Thorlabs IO-4-1220-VLP) was placed between the DFB-DL and the FiberPort to minimize the impact of back-reflections on pump laser stability. The PM fiber directed the pump laser light through a BOA (Thorlabs BOA1210P), which was driven by separate current and temperature controllers (ILX Lightwave LDC-3232 and LDC-3916372). Amplified pump light then traveled via another PM fiber to a fiber-coupled PPLN-WG module with an integrated thermoelectric cooler (HC Photonics, Hsinchu City, Taiwan). The PPLN-WG temperature was controlled by a PID controller (HC Photonics DTSC-20). Fiber-coupled visible light from the PPLN-WG was collimated for characterization and use by a collimation lens package (Thorlabs F110APC-633).

## 4. Fixed-Wavelength Characterization

The system was first characterized for SHG performance and fixed-wavelength operation. To determine the QPM temperature and temperature bandwidth of the PPLN-WG, the intensity of the SHG light was measured with a photodetector (Thorlabs PDA10A2) while the pump laser wavelength remained fixed at 1194.750 nm. This wavelength was verified with an IR wavemeter (Burleigh, Fishers, NY, USA, WA-1000). The temperature of the PPLN-WG was then varied, and the resulting QPM curve is shown in Figure 2b along with a best-fit sinc^2^ profile as described in Section 2.1. The best-fit profile yields a QPM temperature of 329 K and a bandwidth (full-width at half maximum, FWHM) of 3.2 K. To determine the optical frequency bandwidth of the PPLN-WG, the crystal temperature was fixed at 329 K while the wavelength of the pump laser was varied by tuning the DFB-DL temperature. The SHG intensity was again monitored by the same photodetector, and the output optical frequency was measured by using a visible wavemeter (HighFinesse, Tübingen, Germany, WS6-200 UV2). The resulting measurements are shown in Figure 2c, and the best-fit profile yielded a bandwidth of 118 GHz at the second-harmonic wavelength. The deviation from the theoretical sinc^2^ shape in Figure 2b,c may have been due to mild aperiodicity in the manufacturing of the PPLN-WG or pump depletion effects [10,23]. The SHG efficiency of the PPLN-WG was evaluated by fixing the wavelength of the pump DFB-DL at 1194.750 nm and the temperature of the PPLN-WG at 329 K while varying the gain of the BOA. The pump power was measured after the BOA, and the SH power was measured after the PPLN-WG by using fiber-coupled power meters (Thorlabs PM20CH and PM20A, respectively). The resulting measurements are shown in Figure 2d, along with a best-fit of Equation (2). The measured efficiency was η = 109%/W, which is comparable to other studies in the literature employing a PPLN-WG for SHG [12,13,14,16].

**Figure 3:**
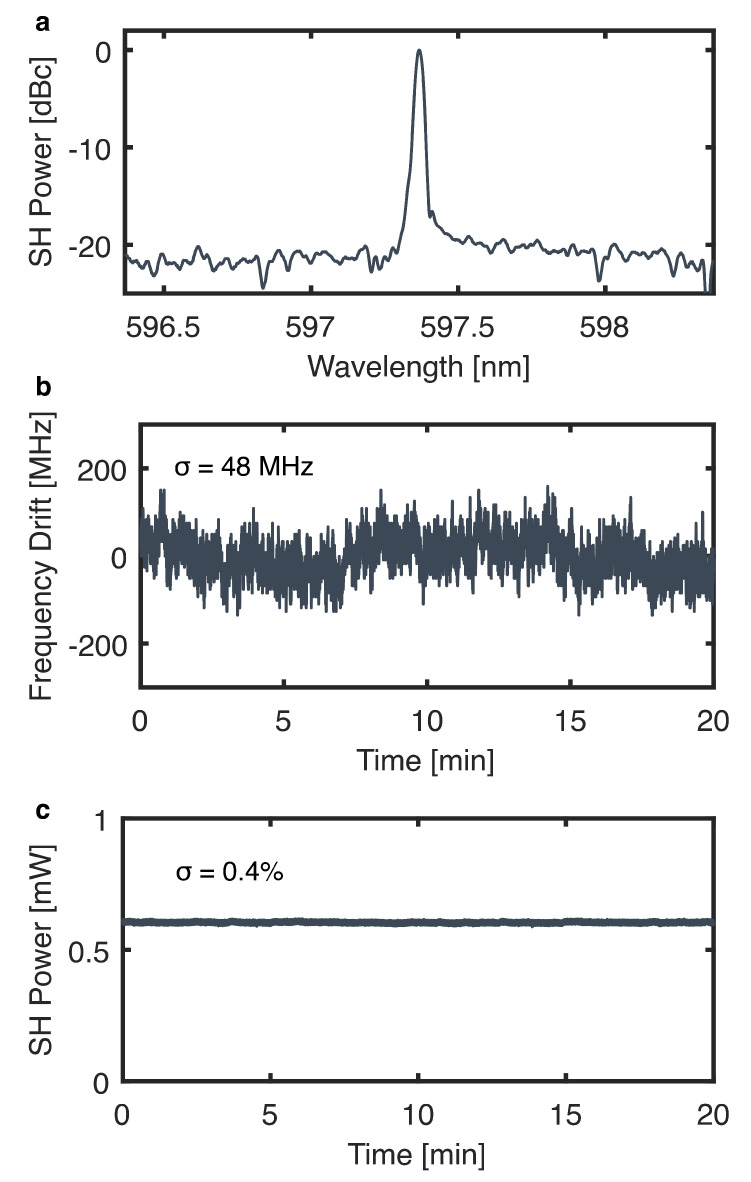
(**a**) Representative output spectrum of the laser system. Long-term optical frequency (**b**) and power (**c**) stability of the system.

The single-frequency operation of the system was confirmed by examining the output in a laser spectrum analyzer (HighFinesse LSA UV2). A representative spectrum is shown in Figure 3a, which clearly indicates single-frequency, narrow-linewidth behavior. The output linewidth fell below the linewidth measurement resolution of the spectrum analyzer (7 GHz) and was also confirmed to be below 0.8 GHz by using a solid YAG etalon (Lightmachinery, Nepean, ON, Canada, OP-3091-98000; see Section 5). The linewidth of the visible output is likely much smaller than this, as it is primarily determined by the linewidth of the pump laser, which is less than 3 MHz [28]. The long-term optical frequency and power stability of the system were assessed by monitoring the wavelength by using the visible wavemeter and the intensity by using a photodetector over a span of 20 min. The resulting optical frequency and power measurements are shown in Figure 3b and Figure 3c, respectively. Over this time period, the system maintained its optical frequency and power to within standard deviations of 48 MHz and 0.4%, respectively. Short-term stability was also assessed, though the measurement rate of the visible wavemeter precluded measurements of optical frequency on sub-millisecond timescales. In terms of intensity, the system exhibited similar short-term and long-term stability. The intensity was typically stable to within a standard deviation of 0.4% over the span of 10 ms, which is a timescale representative of the full course of the shock tube experiments described in Section 6.

## 5. Rapid-Tuning Characterization

**Figure 4:**
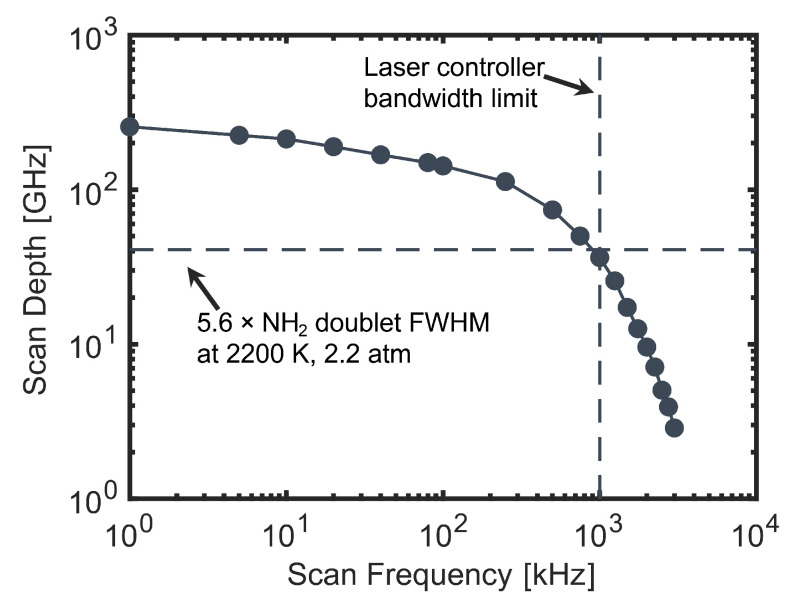
The maximum scan depth for the visible output of the system as a function of the scan frequency.

The system was characterized for rapid-tuning operation in two use cases: SDA and WMS. In SDA, the maximum scan depth (in optical frequency) is of primary concern, as the entire feature must be captured in each scan in order to perform a sensitive lineshape fit. This maximum scan depth was evaluated by sinusoidally scanning the DFB-DL injection current between the lasing threshold and the maximum rated current while adjusting the scan frequency. The scan depth was then measured by using the solid YAG etalon and photodetector mentioned in Section 4. The results of this characterization are shown in Figure 4. The primary factor attenuating the scan depth in this case was the DFB-DL controller bandwidth, which was nominally 1 MHz. A rule of thumb to minimize Voigt fit uncertainty in SDA measurements is to scan 5.6 times the FWHM of the target feature, which is indicated in Figure 4 for the target NH_2_ doublet under representative conditions of 2200 K and 2.2 atm [37]. The present system exceeds this target up to a scan frequency of 900 kHz, corresponding to a measurement rate of 1.8 MHz. This indicates that the system is capable of conducting scanned measurements of NH_2_ with microsecond time resolution, which is a critical capability for the study of rapid, transient processes such as ignition.

**Figure 5:**
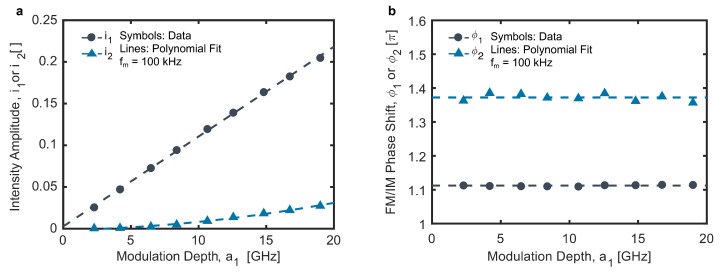
Linear (black) and nonlinear (blue) intensity amplitudes (**a**) and phase shifts (**b**) as functions of the modulation depth at a modulation frequency of 100 kHz. The dashed lines are the polynomial fits to the measured data.

The system was characterized for use in WMS following the procedure described by Li et al. [27]. The resulting WMS model parameters, as defined in Equations (8) and (9), are shown in Figure 5 as functions of the modulation depth for $f_{m}$ = 100 kHz. The measured linear and nonlinear intensity amplitudes, $i_{1}$ and $i_{2}$, are shown in Figure 5a and exhibit typical behavior for a DFB laser. The $i_{1}$ values increase linearly with the modulation depth, while $i_{2}$ increases quadratically. However, what is more notable is the relatively small magnitude of $i_{1}$, especially relative to $i_{2}$. This is likely derived from two effects. First, intensity modulation in the output of the DFB-DL, as driven by the injection current modulation, is dampened by the BOA in the system, which operates near saturation. This reduces $i_{1}$ for the visible output. Second, the efficiency of the SHG process in the PPLN-WG is dependent on the optical frequency of the pump light, as per the QPM optical frequency bandwidth curve shown in Figure 2c. While the QPM frequency bandwidth (118 GHz) is relatively large compared with the modulation depth, even this small variation in SHG efficiency over the span of a modulation period increases $i_{2}$ for the visible output. The accompanying linear and nonlinear frequency modulation/intensity modulation (FM/IM) phase shifts, $\phi_{1}$ and $\phi_{2}$, are shown in Figure 5b as functions of the modulation depth. Again, the present system behaves similarly to a DFB laser. The FM/IM phase shifts are independent of modulation depth and are just above π, which would be the expected linear phase shift at very low modulation frequencies.

## 6. Demonstration

The capabilities of the present system were demonstrated in a series of NH_3_ pyrolysis experiments behind reflected shocks in a shock tube. The goal of these experiments was to demonstrate the utility of the system in four use cases common for DFB lasers as applied to high-temperature spectroscopic and chemical kinetic studies: FDA, SDA, IMS, and WMS. All of these experiments were performed in the Stanford University Flexible Applications Shock Tube (FAST) facility, which has an inner diameter of 14.12 cm. Further details about this facility are available in the literature [38]. The visible laser system was aligned through the shock tube test section in a double-pass configuration via two pairs of sapphire windows and was focused onto a photodetector with a bandwidth of 150 MHz (Thorlabs PDA10A2). A narrow-bandpass spectral filter centered at 600 nm with an FWHM of 25 nm (Edmund Optics, Barrington, NJ, USA, 65-163) was used to reject thermal emission. Signals from the photodetector and a high-speed pressure transducer (Kistler, Winterthur, Switzerland, 603B1) were recorded on a digital oscilloscope at a sample rate of 125 MHz (Pico Technology, St. Neots, UK, 5444D). A mid-IR SDA diagnostic was simultaneously deployed on the shock tube to measure the initial NH_3_ mole fraction. Details on this diagnostic have been previously published [38,39,40]. The shock tube and mixing tank were also passivated by using procedures outlined in a previous publication to minimize the effects of NH_3_ adsorption [38]. Measurements were conducted over a narrow range of conditions for demonstration purposes: 2100 to 2200 K, 2.2 to 2.3 atm, and approximately 2.2% NH_3_ diluted in argon.

### 6.1. Representative Raw Data

**Figure 6:**
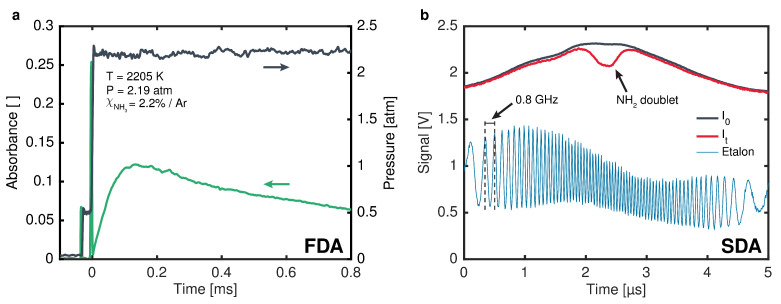
(**a**) Representative pressure (black) and FDA absorbance (green) time histories for post-reflected shock conditions of 2205 K and 2.19 atm and an initial NH_3_ mole fraction of 2.2%. (**b**) Representative raw incident (black) and transmitted (red) intensity for an SDA experiment, along with a representative etalon measurement (blue). Half of a scan period is shown.

A representative pressure time history from a pyrolysis experiment is shown in Figure 6a. These experiments were conducted behind reflected shock waves; thus, two sharp increases in pressure are visible in the time history. The first is the arrival of the incident shock wave, and the second is the arrival of the reflected shock wave. Constant-pressure conditions were maintained for approximately 2 ms for all experiments. However, less than 1 ms was considered for spectroscopic data processing. For FDA experiments, the system was tuned near the peak of the target doublet at an optical frequency of 16,739.900 cm^−1^, as verified by the visible wavemeter mentioned in Section 4. A representative FDA time history is shown in Figure 6a. The measured absorbance trace reflects the processes governing the mole fraction of NH_2_ during the experiment. Following the passage of the reflected shock, the NH_3_ in the test gas begins to break down, generating NH_2_ radicals and leading to an increase in the measured absorbance. These radicals subsequently react further, eventually forming stable N_2_ and H_2_. The balance between NH_2_ production and consumption leads to a peak in the NH_2_ mole fraction and absorbance, after which NH_2_ consumption dominates and the measured absorbance declines.

**Figure 7:**
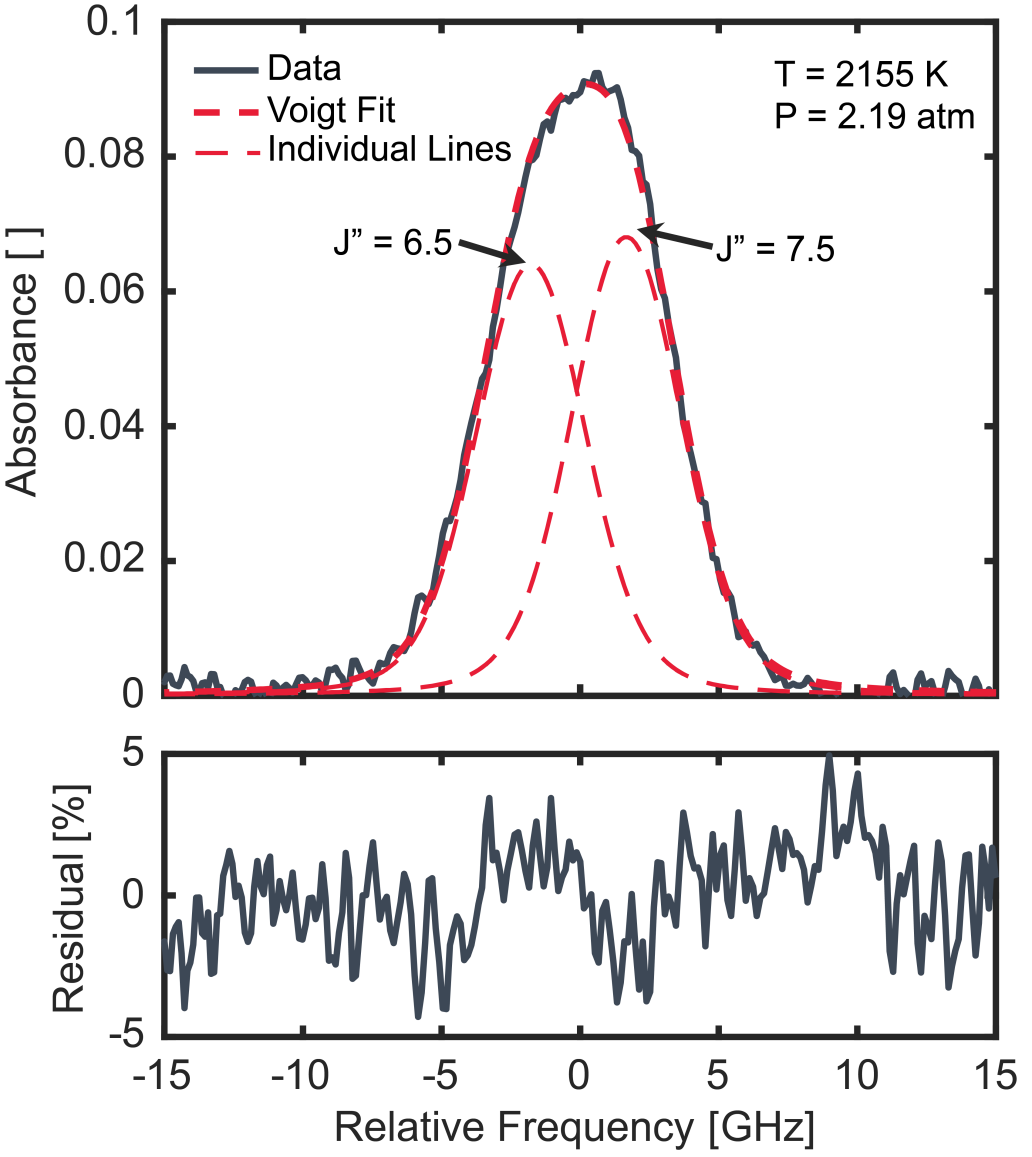
Measured (black) and best-fit (red) absorbance profile and fit residual for a representative SDA experiment under post-reflected shock conditions of 2155 K and 2.19 atm. Individual transitions and total doublet fit are shown as thin and thick red dashed lines, respectively.

In the SDA experiments, the output of the system was scanned over the doublet by sinusoidally scanning the injection current of the DFB-DL at a frequency of 100 kHz. Representative measurements from one half of a scan period are shown in Figure 6b. In this figure, the incident intensity, $I_{0}$, was taken from a scan recorded prior to the arrival of the incident shock and the transmitted intensity, $I_{t}$, was taken from a scan near the time of maximum NH_2_ concentration. The NH_2_ doublet is clearly visible in the center of the figure. The non-sinusoidal behavior of the intensity during the scan is a result of the QPM frequency bandwidth curve, which peaks close to the center of the scan. As in the SDA characterization in Section 5, a solid YAG etalon with a free spectral range of 0.8 GHz was used to determine the relative optical frequency response during each scan, also shown in Figure 6b. A measured absorbance profile from a representative SDA experiment is shown in Figure 7, along with the two individual best-fit Voigt profiles, the overall fit, and the fit residual. While the doublet profile cannot be represented by a single Voigt, the two transitions, indicated by their lower-state J value (J″) in the figure, are quite blended under this condition. Thus, to adequately fit this profile and all SDA profiles measured in this work, the doublet splitting and ratio between the doublet oscillator strengths were fixed by using the values measured by Kohse-Höinghaus et al. [6] (3.44 GHz and 0.93, respectively). The collisional widths of the two transitions making up the doublet were also assumed to be equal. Thus, only two parameters were varied in the fit: a single integrated area and a single collisional width. By using this scheme, fit residuals were consistently within ±5% of the peak absorbance, except for at very early and very late times during shock experiments when NH_2_ concentrations were low.

**Figure 8:**
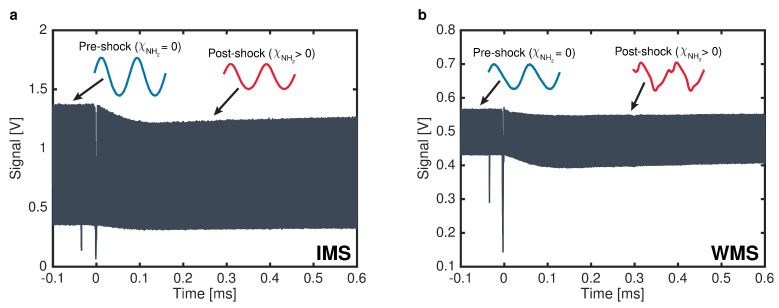
(**a**) Representative raw IMS data during a shock experiment. Pre-shock (blue) and post-shock (red) signals are highlighted, showing the drop in amplitude due to the presence of absorbing NH_2_. (**b**) Representative raw WMS data during a shock experiment. Pre-shock (blue) and post-shock (red) signals are again highlighted, illustrating the presence of the NH_2_ absorption feature following the shock.

For IMS, the injection current of the BOA was sinusoidally modulated at $f_{m}$ = 2 MHz. This enabled the independent modulation of the system output intensity while the optical frequency remained fixed at 16,739.900 cm^−1^, as verified by the visible wavemeter. Representative measured laser intensity from a shock experiment using IMS is shown in Figure 8a. Individual modulation cycles blend together at the timescale shown in the figure, but portions of the pre-shock and post-shock signals are inset in blue and red, respectively, to show finer details of the signals. The measured intensity deviates slightly from a sinusoid, likely due to the combined effects of amplifier saturation and the nonlinear dependence of SHG efficiency on the power output from the BOA, as illustrated in Figure 2d. The only notable difference between the pre- and post-shock signals is a smaller amplitude post-shock caused by the absorption of light by NH_2_ molecules. To calculate NH_2_ time histories from these measured data, digital lock-in filtering at $f_{m}$ was performed with a bandwidth of 500 kHz. The resulting lock-in signal scales approximately with the amplitude of the trace shown in Figure 8a. The average lock-in signal prior to the arrival of the incident shock was taken as the incident intensity ($I_{0}$ in Equation (3)), and the lock-in signal post-shock was taken as the transmitted intensity. ($I_{t}$ in Equation (3)) The Beer–Lambert relation, given in Equation (3), was then used to calculate the NH_2_ mole fraction.

Finally, for WMS experiments, the injection current of the DFB-DL was sinusoidally modulated at $f_{m}=700$ kHz with an optical frequency modulation depth of 5.79 GHz. This modulation depth was selected to maximize the WMS-2*f*/1*f* signal under the target measurement conditions as per the recommendations by Peng et al. [37]. The average optical frequency, or $\bar{\nu }$ in Equation (8), was measured with the visible wavemeter prior to each experiment with the injection current modulation disabled. The optical frequency and intensity tuning of the laser were also characterized prior to each experiment by using the same method as in Section 5. The representative measured laser intensity from a shock experiment using WMS is shown in Figure 8b. As in the IMS data, individual modulation cycles appear blended together, but portions of the pre- and post-shock signals are inset in blue and red, respectively, to illustrate finer details. Once again, the measured intensity deviates from a sinusoid, in this case more significantly than in IMS. This more significant deviation is a direct result of the QPM optical frequency bandwidth of the PPLN-WG, which was not a factor in IMS, as the optical frequency remained fixed when using that technique. The most significant difference between the pre- and post-shock signals is the appearance of periodic absorption features in the latter due to the presence of NH_2_. To calculate NH_2_ time histories from these raw WMS data, digital lock-in filtering with a bandwidth of 500 kHz was used to extract the 1f and 2f signals. The 1f signal scales approximately with the amplitude of the raw voltage trace, as in IMS, and the 2f signal is most sensitive to the peak absorbance of the NH_2_ absorption feature observed post-shock. These 1f and 2f signals were then divided, effectively normalizing out any laser intensity variations, to produce a WMS-2*f*/1*f* signal. The average WMS-2*f*/1*f* signal prior to shock arrival was taken as the background and was subtracted from the WMS-2*f*/1*f* signal post-shock. Background-subtracted WMS-2*f*/1*f* signals were then calculated from Equations (3), (8), and (9) by using the optical frequency and intensity tuning characterizations conducted prior to the experiment, the NH_2_ spectroscopic constants discussed in Section 2.3, and the same lock-in filter as that used on the measured signals. These calculations were performed at the post-reflected shock temperature and pressure of each experiment over a range of NH_2_ mole fractions from 0 to 1000 ppm. The measured NH_2_ mole fraction time histories were then computed by linearly interpolating the simulated mole fraction-dependent WMS-2*f*/1*f* signals at the measured WMS-2*f*/1*f* values.

### 6.2. Time History Measurements

**Figure 9:**
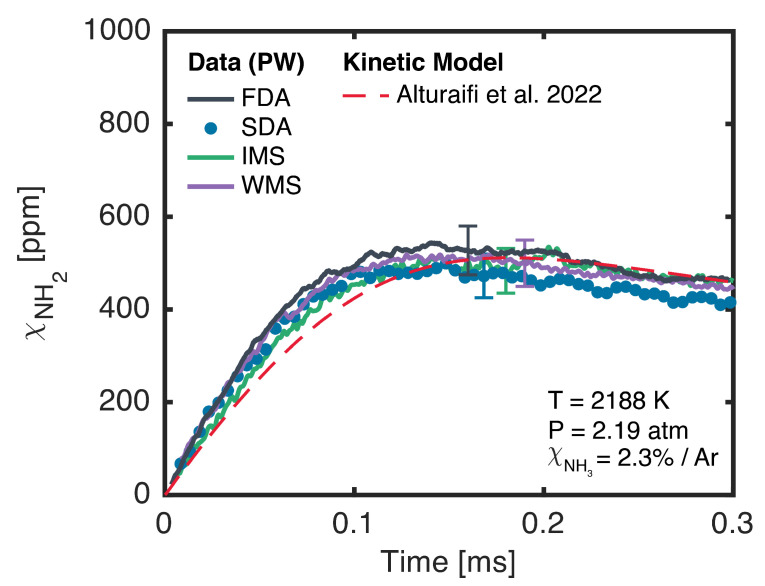
Measured NH_2_ time histories using FDA (black), SDA (blue), IMS (green), and WMS (purple) for average conditions of 2188 K and 2.19 atm with an initial NH_3_ mole fraction of 2.3%. Data are compared to a model published by Alturaifi et al. [42] (red). Error bars are ±1σ uncertainties.

The time histories of the NH_2_ mole fraction during NH_3_ pyrolysis were measured by using all four techniques described in the preceding section. The results are shown in Figure 9 under average conditions of 2188 K, 2.19 atm, and 2.3% initial NH_3_. All four experiments fell within ±25 K, ±0.06 atm, and ±0.1 percentage points of this average. The measured FDA time history shown in Figure 9 was moving-average-filtered to a measurement bandwidth of 500 kHz, which matches the lock-in bandwidths of the IMS and WMS measurements. The SDA time history was moving-average-filtered to a measurement bandwidth of 100 kHz. The error bars shown in Figure 9 represent ±1σ uncertainties on the measurements, which were calculated by using the Taylor series approach to uncertainty propagation [41]. These uncertainties were dominated by the ±10% uncertainty on the oscillator strength as reported by Votsmeier et al. [35]. Time histories from all four techniques agreed within uncertainty, and the small variations between the traces were likely due to small differences in the initial NH_3_ concentration and post-reflected shock conditions. The result of a chemical kinetic simulation using the model from Alturaifi et al. [42] is also shown in Figure 9 for comparison. This simulation was conducted under the average conditions of the four experiments. These time history measurements illustrate the capacity of the present laser system to provide valuable data for the evaluation of high-temperature chemical kinetic models for NH_3_ pyrolysis and oxidation. In this particular example, the measured initial rate of formation of NH_2_ was modestly underpredicted by Alturaifi et al.’s [42] model, but the model accurately captured the peak NH_2_ mole fraction. This agreement in peak NH_2_ is unsurprising, as Alturaifi et al.’s [42] model was validated against peak NH_2_ mole fraction measurements reported by Davidson et al. [33]. The measurement of such time histories over a wider variety of conditions and mixtures will be the focus of future work, with the goal of improving the state of knowledge of high-temperature NH_3_ chemistry.

### 6.3. Lineshape Measurements

**Figure 10:**
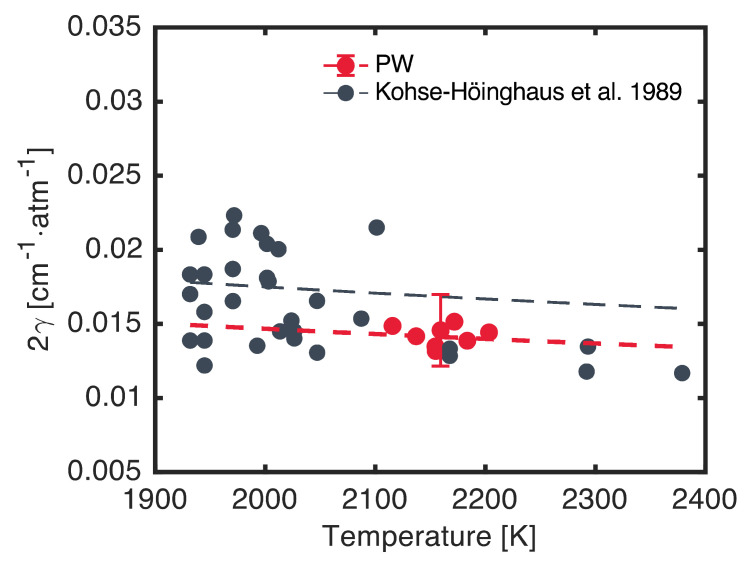
Measured argon broadening coefficient as a function of temperature for the target transitions (red) in comparison to literature data from Kohse-Höinghaus et al. [6] (black). Power-law correlations are shown as dashed lines. Error bars are ±1σ uncertainties.

The lineshape of the target doublet was measured by using both SDA and WMS. The argon broadening coefficients were extracted from the SDA measurements and are shown in Figure 10 as functions of the temperature in comparison to measurements by Kohse-Höinghaus et al. [6]. As discussed in Section 6.1, the collisional widths of the transitions making up the target feature were assumed to be equal, and as such, a single 2$\gamma_{Ar}$ measurement is reported for each experiment. The resulting measurements agree well with previous work by Kohse-Höinghaus et al. [6], though they display lower scatter. The uncertainties in the present 2$\gamma_{Ar}$ measurements, shown as representative ±1σ error bars in Figure 10 and calculated by using the Taylor series method, were dominated by scan-to-scan variation and fit uncertainty. The average ±1σ uncertainty in 2$\gamma_{Ar}$ was ±15%. A power-law correlation fit to the present measurements is also shown in Figure 10. Due to the narrow temperature range considered in the present study, the temperature-dependence exponent in this fit was fixed to $n_{Ar}=0.5$. This is the value recommended by Kohse-Höinghaus et al. [6] and that arises from a hard-sphere collision approximation [24]. The resulting reference broadening coefficient, $2\gamma_{Ar}$ (2000 K), was 0.0147 cm^−2^·atm^−1^. This value is smaller than the value of 0.0175 cm^−2^·atm^−1^ recommended by Kohse-Höinghaus et al. [6] and plotted in Figure 10. However, the value recommended by Kohse-Höinghaus et al. [6] does agree with the present broadening coefficients within measurement uncertainty. Future efforts will focus on extending and refining these broadening coefficient measurements to provide an updated measurement of $n_{Ar}$.

**Figure 11:**
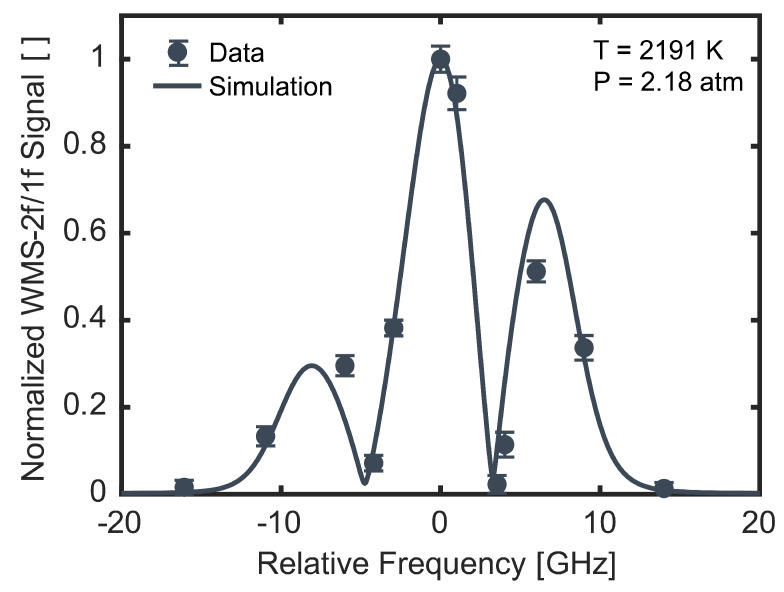
Measured WMS-2*f*/1*f* lineshape (points) of the target NH_2_ doublet under nominal conditions of 2191 K, 2.18 atm, and 2.2% initial NH_3_ compared with a simulated lineshape (line). Error bars represent ±1σ standard deviations of the measured signals.

To map out the WMS-2*f*/1*f* lineshape of the feature, WMS experiments were repeated with different average optical frequencies. These experiments were performed under average conditions of 2191 K, 2.18 atm, and 2.2% initial NH_3_. All experiments fell within ±16 K, ±0.04 atm, and ±0.1 percentage points of this average. The resulting peak WMS-2*f*/1*f* signals are shown as functions of the optical frequency in Figure 11, normalized by the signal at the center of the feature. The error bars represent ±1σ standard deviations of the measured signal in the vicinity of the peak. The measurements are shown in comparison to a simulated WMS-2*f*/1*f* lineshape. The measurements agree well with the simulated lineshape, though disagree slightly near ±6 GHz. Improved measurements of the argon broadening coefficient may lead to closer agreement between measured and modeled WMS-2*f*/1*f* lineshapes. Nevertheless, this measured WMS-2*f*/1*f* lineshape illustrates the utility of the present system for use in WMS diagnostics, which may enable future development of compact, fiber-coupled, narrow-linewidth, visible laser systems for WMS measurements in practical devices, similar to how near- and mid-IR DFB lasers are often deployed [1].

## 7. Conclusions

This work presented the design, characterization, and demonstration of a rapidly tunable laser system for the generation of narrow-linewidth visible light at 597.4 nm. The system was based on an amplified DFB-DL which was frequency-doubled in a PPLN-WG. Because of the high SHG efficiency of the PPLN-WG (109%/W), a single-pass configuration was sufficient to achieve up to 8 mW of output power without the use of an optical cavity. Thus, the mode-hop-free rapid-tuning capabilities of the pump DFB-DL were preserved in the visible output, resulting in a large scan depth of 36.4 GHz in optical frequency demonstrated at a scan rate of 1 MHz. This output also exhibited single-frequency operation with excellent stability; optical frequency and power were maintained within standard deviations of 48 MHz and 0.4%, respectively, over 20 min. The rapid-tuning capabilities of the system were characterized for use in both SDA and WMS experiments. Finally, the laser was demonstrated for use in shock tube studies of NH_3_ pyrolysis, wherein it was used to measure NH_2_ concentration by targeting a doublet transition at 16,739.90 cm^−1^. These measurements were conducted by using the laser system in four modes: FDA, SDA, IMS, and WMS. This extensive demonstration emphasizes the versatility of systems based on DFB-DLs doubled in PPLN-WGs and illustrates that many of the techniques used with standard DFB lasers can be applied to such systems. This work opens the door for the development of rapidly tunable laser systems to probe the high-temperature spectroscopic and kinetic behavior of other species that are active in the visible-wavelength range, including transient radicals.

## Data Availability

The raw data supporting the conclusions of this article will be made available by the authors upon request.
